# Form, synapses and orientation topography of a new cell type in layer 6 of the cat’s primary visual cortex

**DOI:** 10.1038/s41598-022-19746-9

**Published:** 2022-09-14

**Authors:** Mohit Srivastava, Cintia Angel, Réka Eszter Kisvárday, Zsolt Kocsis, András Stelescu, Petra Talapka, Zoltán Kisvárday

**Affiliations:** 1 ELKH-DE Neuroscience Research Group, Department of Anatomy, Histology and Embryology, University of Debrecen, Debrecen, Hungary; 2 Department of Anatomy, Histology and Embryology, Faculty of Medicine, University of Debrecen, Debrecen, Hungary; 3 Department of Neuroanatomy and Molecular Brain Research, Faculty of Medicine, Ruhr-Universitat Bochum, Bochum, Germany

**Keywords:** Neural circuits, Visual system, Striate cortex

## Abstract

Here we report the morpho-functional features of a novel type of deep-layer neuron. The neuron was selected from a large pool of intracellularly labelled cells based on the large cell body, numerous spine-free dendrites with an overall interneuron morphology. However, the axon gave off long-range axons up to 2.8 mm from the parent soma in layers 5/6 before entering the white matter. The boutons were uniformly distributed along the axon without forming distinct clusters. Dendritic length, surface area and volume values were at least 3 times larger than any known cortical neuron types with the exception of giant pyramidal cells of layer 5. Electron microscopy of the boutons revealed that they targeted dendritic spines (78%) and less frequently dendritic shafts (22%). Nearly half of the postsynaptic dendrites were immunopositive to GABA. Superimposing the axonal field on the orientation map obtained with optical imaging showed a preponderance of boutons to cross-orientations (38%) and an equal representation of iso- and oblique orientations (31%). The results suggest an integrating role for the layer 6 stellate neuron which projects to a functionally broad range of neurons in the deep cortical layers and to other cortical and/or subcortical regions.

## Introduction

In the visual cortex, excitatory neurons are glutamateergic^1^ and represent the dominant cell population in all cortical layers except layer 1. The excitatory neuron population shows typically a pyramidal morphology although, in layer 4, spiny stellate and star-pyramidal cell morphology is also common^2–4^. Another characteristic feature of excitatory neurons is that their dendrites bear spines each representing a postsynaptic surface mainly to excitatory inputs arriving either from nearby excitatory neurons or from neurons of other cortical/subcortical areas. On the other hand, there is a large variety of non-spiny or smooth dendritic neurons^5,6^ which are inhibitory in nature and contain the neurotransmitter GABA^7^. Inhibitory neurons receive input synapses on the dendritic shaft that, from the point of view of signal integration, differs radically from that of spine-bearing dendrites. Another difference between the two basic neuron classes regards their synaptic connections with the postsynaptic targets. Excitatory neurons establish type I or asymmetric synapses with the postsynaptic targets whereas inhibitory neurons work via type II or symmetric synapses^8,9^. Furthermore, a large proportion of excitatory neurons are projection neurons, i.e. they send out the axons into the white matter in order to reach other cortical or subcortical regions. Conversely, inhibitory neurons have been considered as local cells (interneuron) without obvious axonal projections into the white matter^10^. In addition to the above neuron classification scheme more recent schemes use connectivity patterns and quantitative morphology (cell body, dendrites and axons) together with biophysical and biochemical parameters^11,12^ as well as information on proteomics and gene expression^13^. While ideally all criteria should be used for neuron classification in most cases only a limited set of the parameters can be obtained.

Here, we report a serendipity discovery of a new neuron type in layer 6 of the primary visual cortex which, intriguingly, shares morphological features of excitatory and inhibitory neuron types. The layer 6 stellate neuron revealed a smooth dendritic morphology, however, its functional orientation topography and synaptic connectivity displayed characteristics of the pyramidal neuron population. Previous anatomical studies using the Golgi-impregnation method took a brief note about the existence of giant stellate neurons with horizontal axons in the fifth layers of the human postcentral gyrus as well as deep layers of the primary visual cortex in the cat and dog without providing any further knowledge^14^. Later studies did mention the existence of intrinsic neurons with smooth dendrites, however, none of them matched the characteristics of the L6 neuron described here^15,16^. Therefore, it is deemed that the L6 smooth dendritic stellate neuron represents a new type.

## Results

### Light microscopic features of the L6 smooth dendritic stellate cell (L6SC)

**Figure 1:**
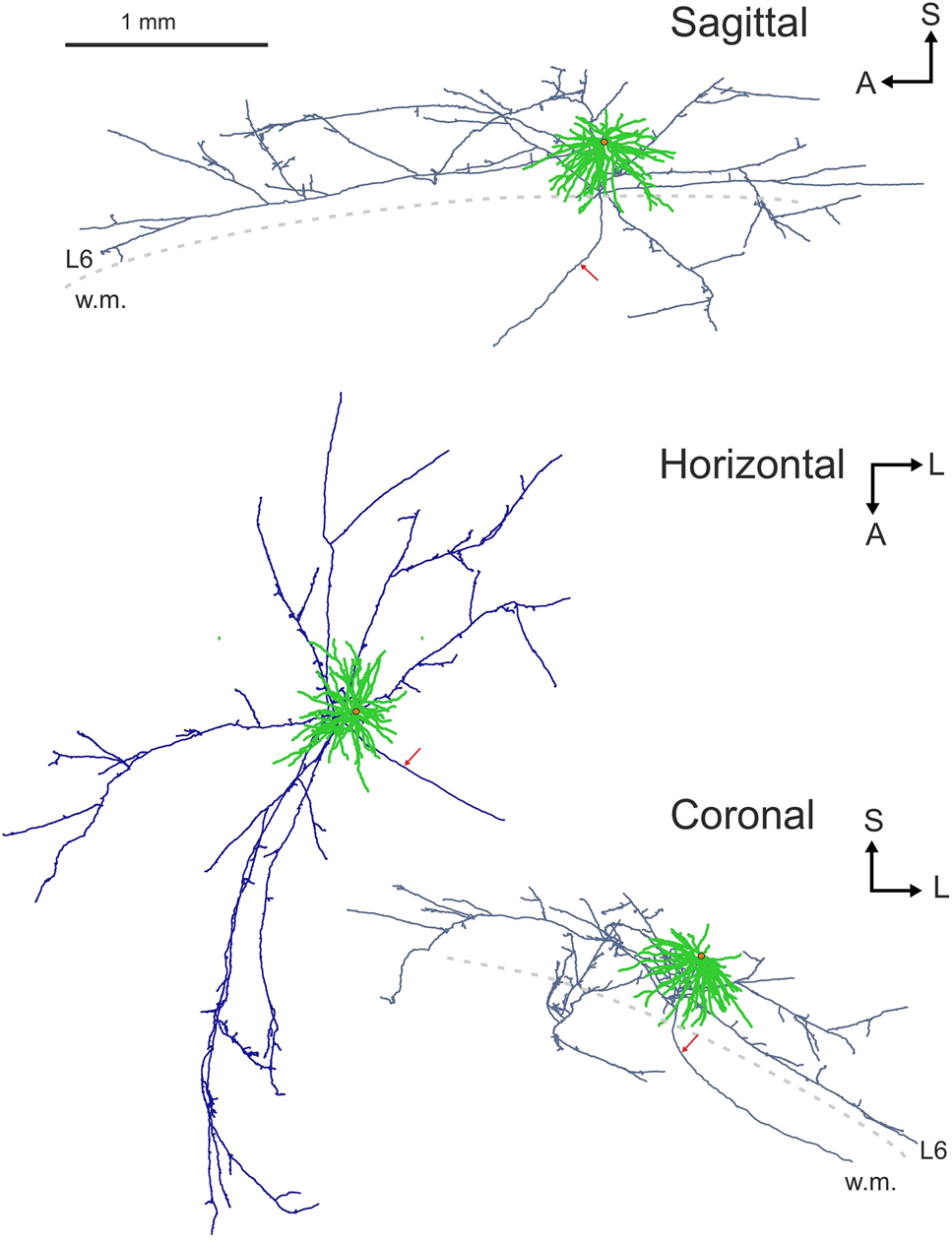
3D-reconstruction of axons (in blue) and dendrites (green) viewed from cardinal anatomical planes (sagittal, horizontal, coronal). Red dot indicates the cell body and red arrow points to the main axon which entered the white matter (w.m.). Drawings were created using the software package Neurolucida (v.8.26, MBF Bioscience, https://www.mbfbioscience.com). *A* anterior, *L* lateral, *S* superior. Scale bar: 1 mm.

**Figure 2:**
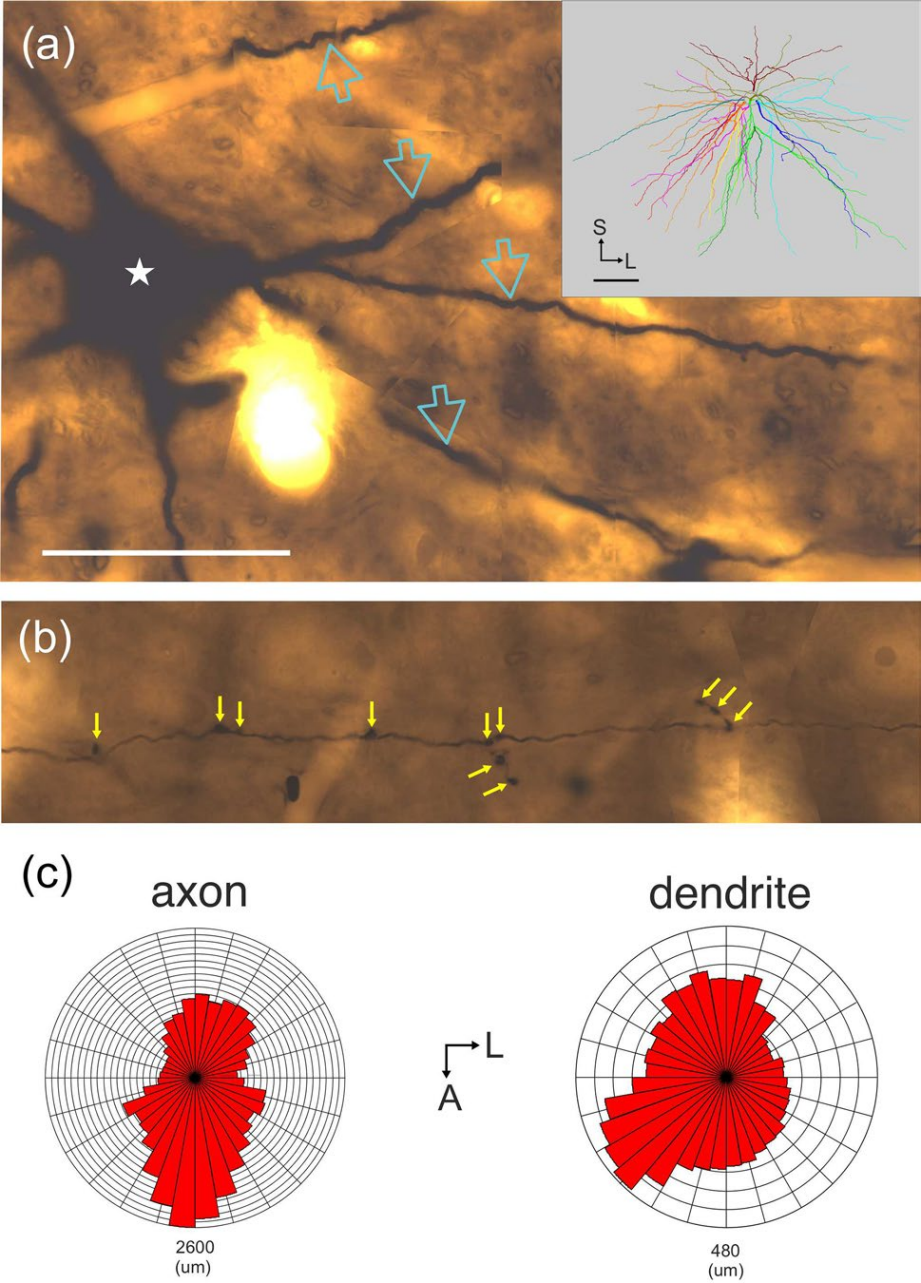
(**a**) High-power LM picture of the soma (star) and the dendrites (arrows) of L6SC. Note that the large cell body (diameter: 29.7 µm) emits thick, spine-free dendrites. Inset shows the 3D-reconstruction of the dendritic field where each dendrite including the daughter branches has different colors, respectively. It is notable that for most of the dendrites the branch points are located proximal to the soma origin followed by a long tapering course radiating out in all directions (drawing was created using the software package Neurolucida (v.8.26, MBF Bioscience, https://www.mbfbioscience.com). (**b**) LM montage of boutons (arrows) along the axon of L6SC. Both *en passant* and *bouton terminaux* are marked by arrows. (**c**) Polar histograms show the axonal (left) and dendritic (right) fields in the horizontal plane. *A* anterior, *L* lateral, *S* superior. Scale bars (**a**,**b**): 50 µm, inset: 0.5 mm.

The cell type described here has a large, round-shaped cell body and numerous, long tapering dendrites which emerged in all directions (Fig. 2a). The cell body was found in the middle of layer 6 and had 29.7 µm in diameter (Table 1). The dendrites were confined largely to L6 and only a few entered the bottom of L5 and others the white matter (Fig. 1). No dendritic spines were observed at all although a few thorny processes could be occasionally present. The dendritic field had a maximum radius of 480 µm in the horizontal plane (Fig. 2c). The total length of the dendrites, their surface area and volume were also determined (Table 1). In this respect, a subset of the basic morphological parameters was compared with those of 3D-reconstructed neurons available in the literature. Accordingly, the total length of dendrites and axons, total dendritic surface area and volume were selected for the sake of simplicity (Table 2). The parameter values showed that L6SC exceeded more than two times the respective values of all other neuron types examined quantitatively. For a direct comparison of the light microscopic phenotype of L6SC with other smooth dendritic neurons, 10 traced and reconstructed neurons of the inhibitory basket cell type were selected and their somata and dendrites are displayed in Suppl. Figs. 1 and 2.

**Table 1:** Basic morphological parameters of L6SC.

Name	Qty	Nodes (Nr.)	Ends (Nr.)	Diameter (µm)	Total length (µm)	Total surface (µm^2^)	Total volume (µm^3^)	Convex hull 3D		Convex hull 2D	
								Volume (mm^3^)	Surface (mm^2^)	Area (mm^2^)	Perimeter (mm)
Soma	1	–	–	29.7	–	2769	13,710	–	–	–	–
Dendrite	14	50	64	–	16,905 mean: 1207	60,257 mean: 4303	20,275 mean: 1447	0.1020	1.1760	0.3386	2.242
Axon	1	606	607	–	38,328	70,587	24,101	3.7480	18.2114	6.8227	10.612

Note that data are corrected for tissue shrinkage. Convex Hull analysis measures the size of the dendritic/axonal field by interpreting the branched structure as a solid object, i.e. a convex polygon is generated by connecting the tips of the distal dendritic/axonal branches. For the 2D analysis, the L6SC was viewed in the tangential plane (parallel to the pial surface).

**Table 2:** Comparison between basic parameters of L6SC and other neuron types in V1.

Cell category	Axon	Dendrite		
	Axon length (µm)	Dendrite length (µm)	Dendrite surface (µm^2^)	Dendrite volume (µm^3^)
L6 stellate cell	38,328	16,905	60,257	20,275
Excitatory neuron types	23,093	7483	20,492	6416
Inhibitory neuron types	39,239	4676	11,931	3100

Comparison between morphological parameters of L6SC with those of previously reconstructed excitatory (L2/3 pyramidal neurons, n = 9^19^; L4 spiny stellate and star-pyramidal neurons, n = 18^20^; L6 pyramidal neurons, n = 26^21^) and GABAergic L3–L4 (basket cells, n = 12^59,60^) neuron types. The L6 stellate neuron has the longest dendritic length (> 2 times) and the largest dendritic surface/volume ratio (> 3 times) compared with any other types published so far. The table displays numerical values for each neuron category.

The main axon of the L6 stellate neuron emerged from the lower part of the cell body and emitted a few recurrent branches before entering the white matter (Fig. 1). The recurrent branches radiated out parallel to the cortical plane and occupied a 4.66 × 2.84 (13.23) mm^2^ area elongated antero-posteriorly (Fig. 2c). The axonal field was largely confined to L6 but a few collaterals were present in L5, though without entering L4. The main axon and those proximal branches were thick indicating the presence of myelin under the light microscope. Most of the distal collaterals were, however, thin indicating the absence of myelin sheath (Fig. 2b). In this regard subsequent electron microscopy could verify the LM observations (see below). Regarding thin axon collaterals, they emitted altogether 1367 boutons of two morphological types: *en passant* (n = 1192) on the one hand and *bouton terminaux* (n = 175) on the other.

### Clustering of the axon

**Figure 3:**
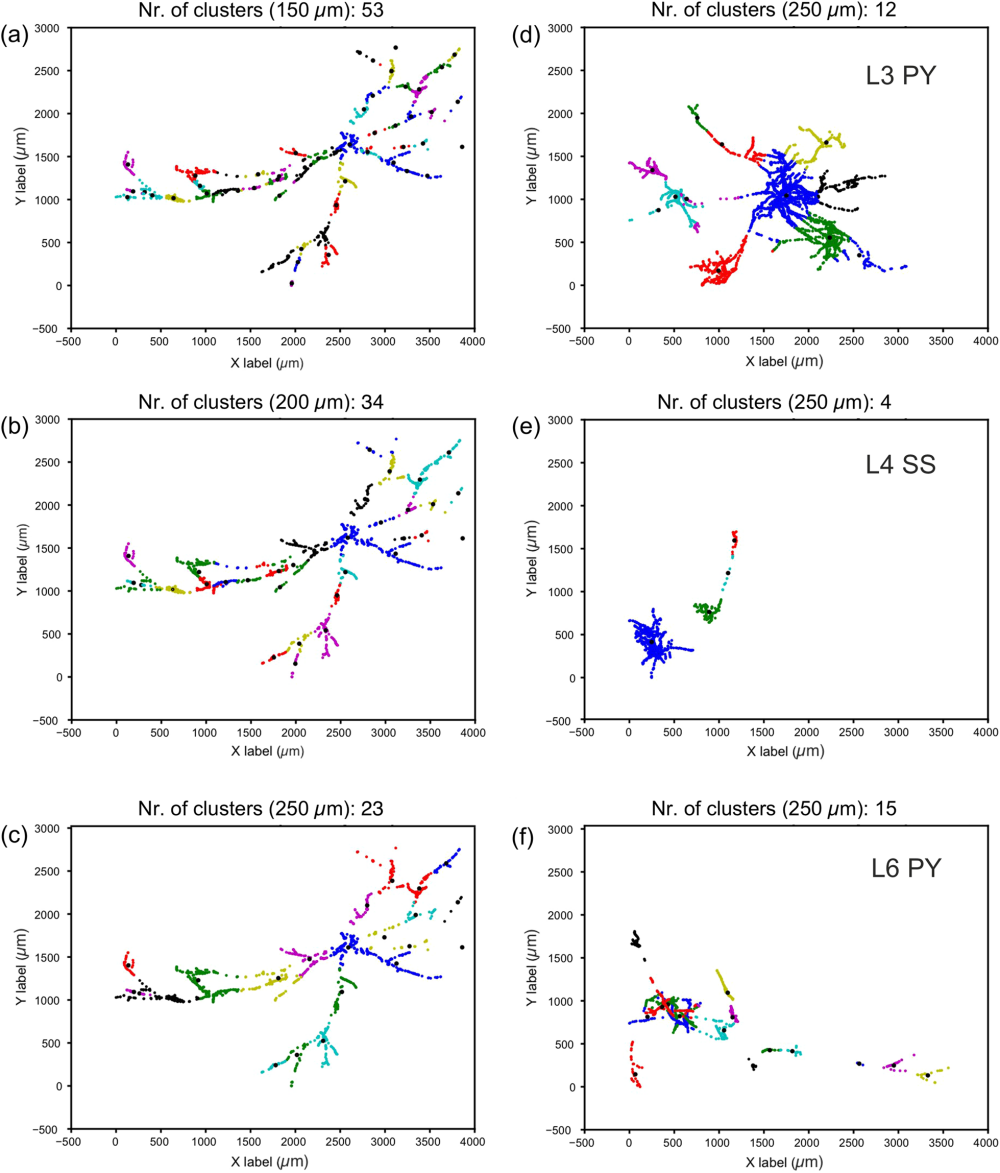
Cluster analysis of the L6SC boutons (**a**–**c**) and comparison with that of known spiny (excitatory) neurons type (**d**–**f**). For L6SC, the mean-shift algorithm produced different number of clusters as a function of kernel size (in parenthesis). Smaller kernel size produced larger number of clusters (**a**) as opposed larger kernel size which produced smaller number of clusters (**b**,**c**). Bouton clustering depends on lateral extent and spatial organization. Using the same kernel size (250 µm) applied in panel (**c**) for L6SC, the exemplary L3 pyramidal cell axon (**d**), L4 spiny stellate axon (**e**), and L6 pyramidal cell axon (**f**) returned 12, 4, and 15 bouton clusters, respectively. For clarity, clusters are shown in color and their centers indicated with small black circles (data were plotted using SpyderIDE 5.1.5, https://www.spyder-ide.org, for further details, see Results).

Another important aspect that was analyzed regards the spatial organization of the L6 axon whether it makes a clustered distribution pattern. It should be noted that clustering of long-range axons is a common feature chiefly in the supra-granular layers of the cortex, but little is known about clustering of axons in the deep layers (for review, see^17^). Therefore, we investigated and compared the clustering character of the L6SC axon with long-range axons of other neuron types as exemplified in Fig. 3. The L6 stellate neuron provided a linear-like distribution of the boutons along the axon without any spatially distinct clustering. In order to back up the qualitative observation, several cluster analysis methods were used of which the mean-shift algorithm was employed^18^. This algorithm returned bouton clusters of different number and size depending on the applied kernel dimension (width). Figure 3a–c show the results using 150, 200, and 250 µm kernel size generating 53, 34, and 23 bouton clusters, respectively. The resulting cluster numbers are in line with our a priory expectation, that the axon of L6SC is organized mainly linearly, for which, decreasing the kernel size produces proportionally more and smaller bouton clusters. In this regard, one should note that the number of bouton clusters depends also on the spatial extent of the axon. For demonstrating the latter, we used 250 µm kernel size for the axons of three representative neuron types taken from the literature: layer 3 pyramidal neuron^19^, layer 4 spiny stellate cell^20^, and layer 6 pyramidal cell^21^. All three neuron types possessing spatially less extensive axons than the giant L6 stellate axon gave rise to fewer bouton clusters by applying the same parameters (Fig. 3d–f).

### Electron microscopy

Light microscopy of the L6 stellate neuron revealed controversial features regarding the presence of smooth dendrites which is a characteristic feature of GABAergic interneuron versus the main axon entering the white matter which is a characteristic feature of excitatory projection neuron in the cortex. Therefore, electron microscopy was carried out for representative parts of the cell body, dendrites and the axon (boutons). Since an important aspect was to unravel the 3D configuration and type of the postsynaptic structures long section series were collected and examined. In addition to this, for identifying presumed inhibitory structures, postembedding GABA-immunohistochemistry was carried out on every 10–20th section using the immunogold technique.

**Figure 4:**
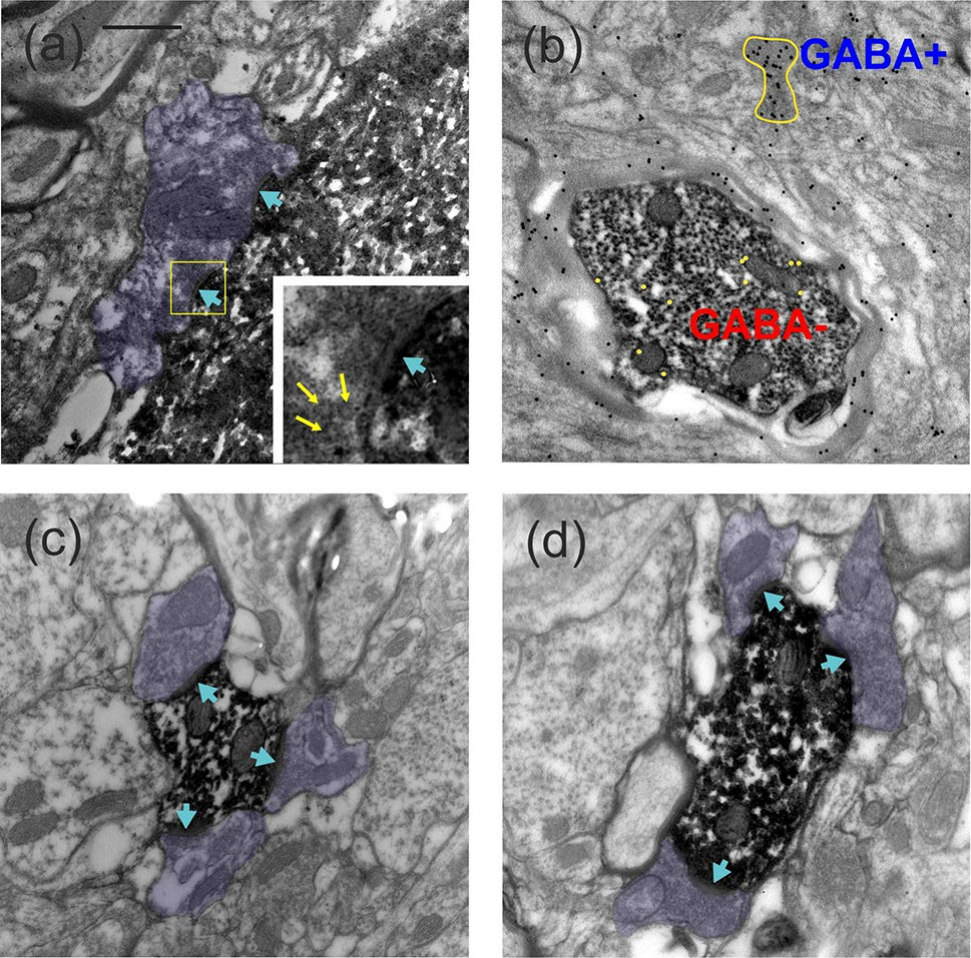
Electron microscopy of the cell body, the main axon and dendrites of the L6 stellate neuron. (**a**) The cell body received putative type I synaptic contacts from a large bouton (purple) providing multiple active zones (blue arrows). Inset shows an enlarged view of the framed area where yellow arrows point to large round-shaped vesicles typical of excitatory synapses. (**b**) The section containing the main myelinated axon was covered and subjected to postembedding GABA-immunogold reaction. The axon was negative to GABA as indicated by the low density of gold-nanoparticles (substituted with 11 yellow dots for better visibility) as opposed to a nearby GABA-immunopositive bouton of unknown origin (yellow contour) which contained a high density of gold-nanoparticles (25 black dots). Note that the axon has an area more than 10 times larger than the bouton. (**c**,**d**) Dendrites of the L6 stellate neuron received numerous synapses (arrows) from a population of morphologically similar boutons (purple). Scale bar (**a**–**d**): 0.5 µm.

The cell body received a large number of synapses from a diverse population of boutons. Some of the large boutons established multiple active zones as shown in Fig. 4a. These axo-somatic synapses contained large round vesicles (inset of panel a) which is typical for excitatory type of synapses^22^. Unfortunately, the dense DAB deposit of the intracellular label did not always allow unequivocal identification of the full extent of postsynaptic thickening due to spilling over. In most of the cases, however, the postsynaptic thickening could be determined unambiguously for which the presence of neighboring type II synapses was helpful.

The main axon was investigated a few µm away from the cell body as well as towards the white matter confirming the LM observation as being myelinated (Fig. 4b). Major axon collaterals were also myelinated with the exception of terminal branches. Serial sectioning of the dendrites revealed a rich synaptic coverage of unknown origin for which two examples are shown in Fig. 4c,d. The boutons of the above axo-dendritic synapses contained densely packed round shape vesicles. Despite the DAB end-product, the postsynaptic thickening could be recognized as a thick band. Inferred from this most of the dendritic input to the L6 stellate neuron was likely to be excitatory.

**Figure 5:**
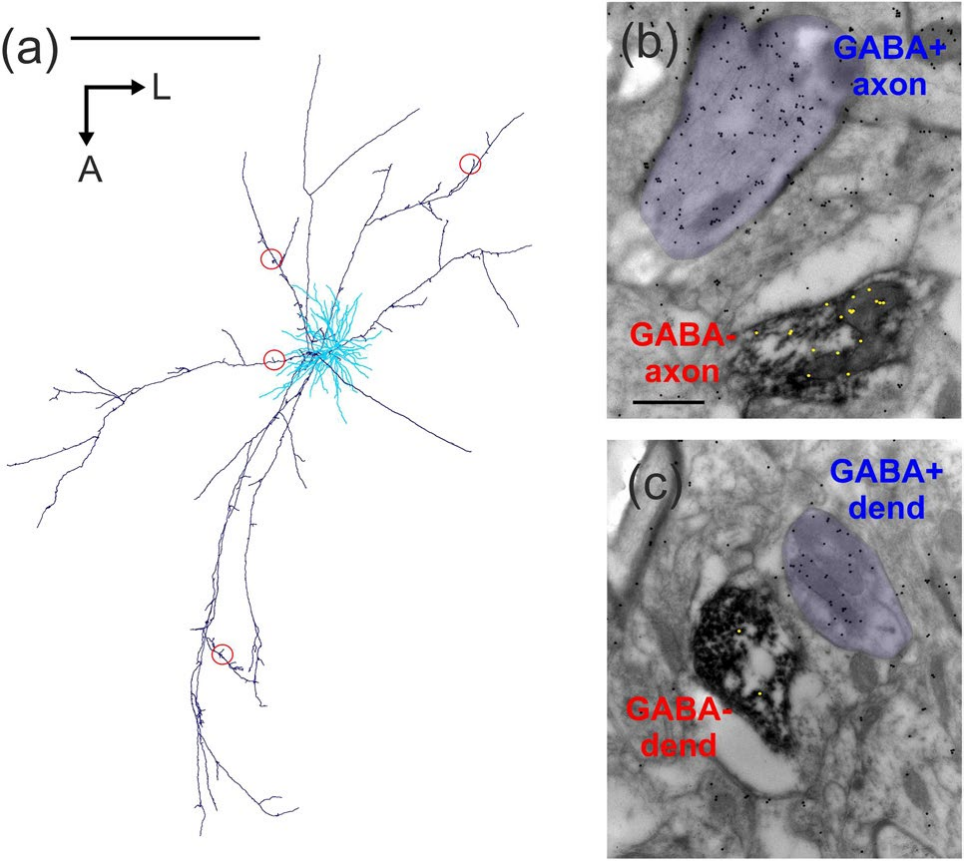
(**a**) Red circles show 4 regions of the L6SC axon (dark blue) which were subjected to EM analysis. The dendritic field is marked in light blue (drawing was created using the software package Neurolucida (v.8.26, MBF Bioscience, https://www.mbfbioscience.com). Exemplary images show postembedding GABA-immunostaining of an axon segment (**b**) and a dendrite (**c**). In (**b**) the DAB labelled L6SC axon shows GABA-immunonegative reaction (the 17 gold nanoparticles were substituted with 17 yellow dots for better visibility) while the other axon of unknown origin (purple) is GABA-immunopositive as represented by the nearly 5 times higher density of gold particles above the axoplasm. (**c**) DAB labelled dendrite of L6SC which showed GABA immunonegativity while the other dendrite (purple shade) of unknown origin was immuno-positive to GABA. Scale bars (**a**): 1 mm, (**b**,**c**): 0.5 µm.

The overall distribution of postsynaptic structures is characteristic for each neuron type (e.g.^23^). For the analysis of the L6SC axon and the postsynaptic structures, tissue samples (EM block) were taken from four sites representing proximal and distal locations within the axonal field width (Fig. 5a). From each site serial sections containing identified axon segments and a total 66 boutons were collected using the correlated LM/EM approach. Postembedding GABA-immunostaining showed that similarly to the main axon non-myelinated axons and dendrites were invariably immunonegative (Fig. 5b,c).

**Figure 6:**
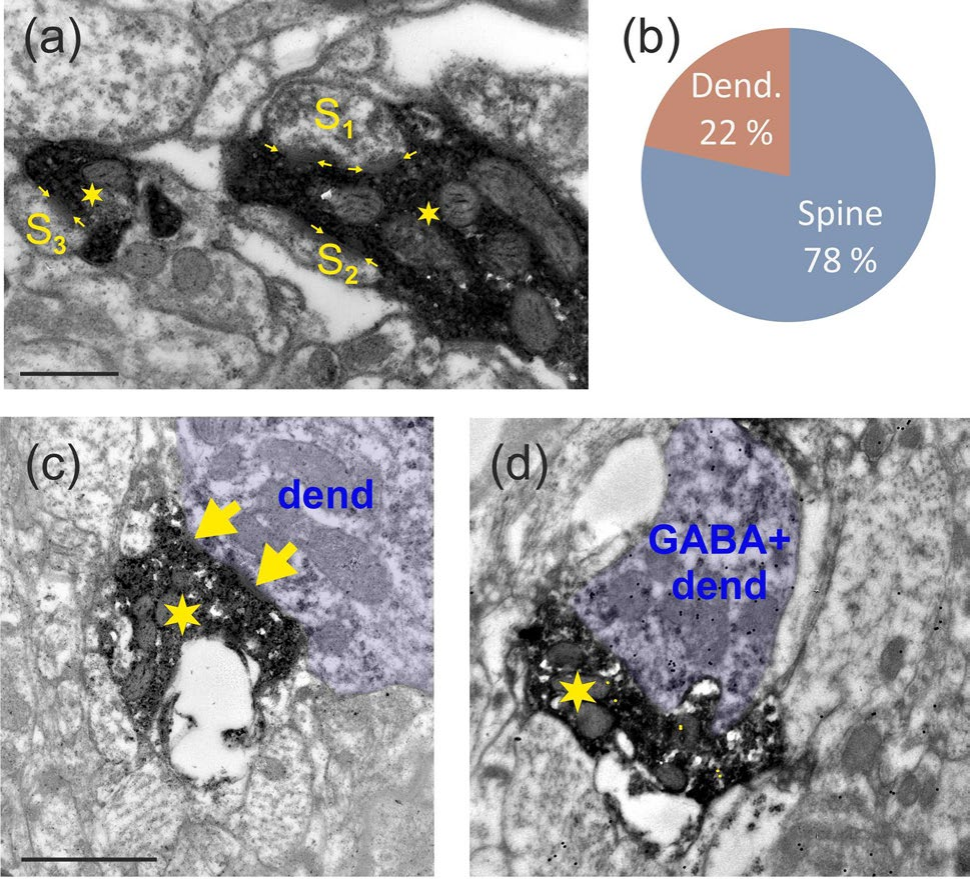
Postsynaptic targets of L6SC. (**a**) L6SC boutons (star) established type I synapses with the head of dendritic spines (S1–3). Many of the synaptic contacts formed discontinuous active zones (small arrows). (**b**) Overall distribution of target structures postsynaptic to L6SC boutons (n = 66). (**c**,**d**) Dendritic shafts (purple shade) were targeted by L6SC boutons (stars), although less frequently than dendritic spines. In (**c**) a mitochondria-rich dendrite (dend) is seen postsynaptic to L6SC (arrow). A few sections away, the same dendrite was tested for GABA-immunostaining (**d**) and showed a positive reaction as indicated by the accumulation of gold-nanoparticles above the bouton. Note that the presynaptic L6SC bouton had GABA-immunolabelling only at the background level (6 yellow dots). Scale bar (**a**): 0.5; (**c**,**d**),: 1 µm.

The main postsynaptic targets were dendritic spines (n = 65) and to a lesser extent dendritic shafts (n = 18). Most of the postsynaptic spines received complex synapses from L6SC by forming discontinuous active zones as illustrated for a spine (S2) in Fig. 6a. It is notable that the same L6SC bouton could establish synapse with more than one structure. Indeed, 17% of the boutons formed contacts with two structures and 5% with three structures, typically with dendritic spines which adds up to a total of 83 postsynaptic structures established by the 66 boutons. Thus, the overall proportion of postsynaptic spines vs dendrites was 78% and 22%, respectively (Fig. 6b). Among the dendrites being postsynaptic to L6SC, four out of nine dendrites proved to be immunopositive for GABA as exemplified in Fig. 6c,d. These immunohistological data indicated that inhibitory as well as excitatory neurons are among the targets of L6SC.

**Figure 7:**
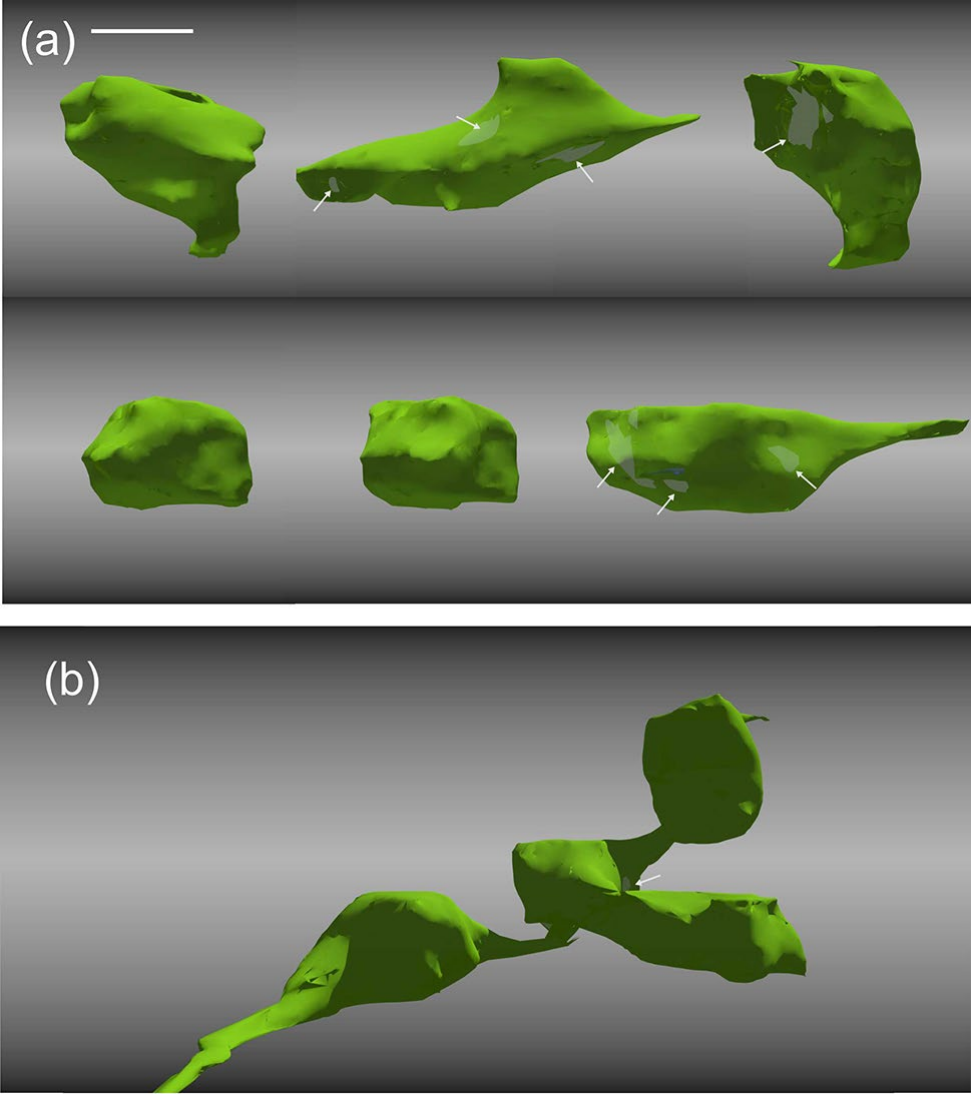
3D-rendering of boutons either in isolation (**a**) or in continuation with each other (**b**). The active zone areas are visible only for some of the boutons (light green) from these perspectives (arrowed for clarity). The 3D-images were created using Blender 2.92 software (GNU General Public License, https://www.blenderguru.com/). Scale bar (**a**,**b**): 1 µm.

For some boutons, the quality of serial EM sections allowed full morphological reconstruction in 3D and in this way to determine their surface area, volume and synapse area (active zone). 3D-rendering of the boutons either in isolation or in continuation is shown in Fig. 7a and b, respectively. The surface and volume measurements as well as the area of the active zone dimensions of the boutons are summarized in Table 3.

**Table 3:** 3D-parameters of L6SC boutons.

L6SC boutons	Volume (µm^3^)	Surface (µm^2^)	Active zone area (µm^2^)
Average (n = 22)	1.48	7.36	0.21
SD	0.68	2.63	0.14

Note that the data is corrected for tissue shrinkage.

### Orientation topography of boutons

**Figure 8:**
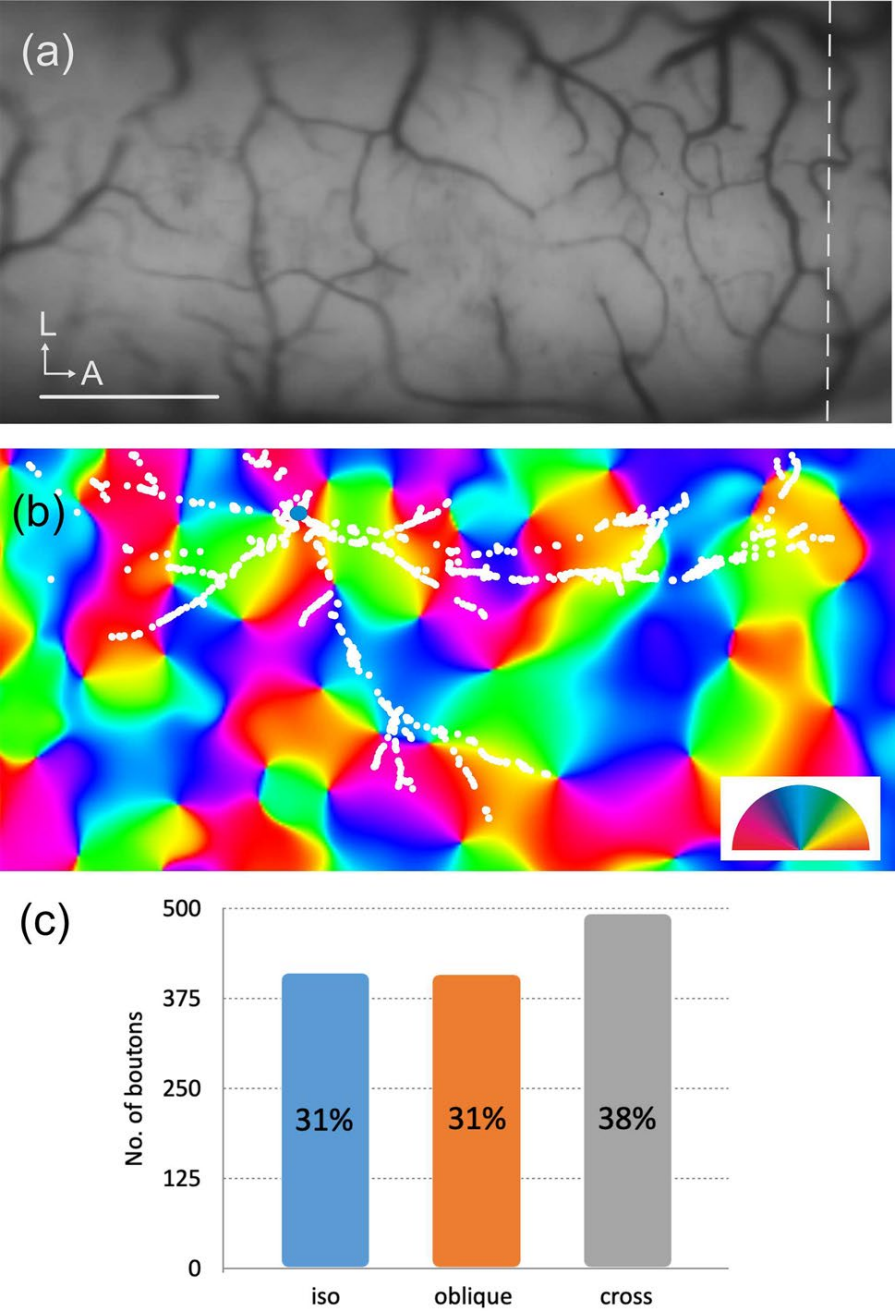
(**a**) Green-image shows the surface vascular pattern used for aligning the Neurolucida reconstruction of L6SC with the orientation map displayed below. Broken line indicates the ear-bar zero position. (**b**) Orientation map overlaid with boutons of L6SC. Only a subset of boutons (87%, white spots) could be taken into account being located within the imaged cortical region. The cell body (blue spot) was found in a region where many orientations converged as can be seen by the rapid change of colors according to the orientation scheme. (**c**) The graph shows the distribution of orientation preferences of boutons simplified as iso-, oblique- and cross-orientation with respect to the orientation preference at the soma location. Most boutons preferred cross-orientation (38%), while iso- and oblique orientations were equal (31% each). *L* lateral, *A* anterior, Scale bar (**a**,**b**): 1 mm.

The L6SC was labelled in a region that had been mapped for orientation using intrinsic signal optical imaging. Orientation topography could be determined for 87% of the boutons since the remaining boutons fell outside of the imaged region (Fig. 8a). Around the cell body location a broad range of orientations could be seen near to a pinwheel position. As a consequence the orientation distribution of the boutons was also broad and did not peak at all at the orientation value of the cell body location (Fig. 8b). For a better interpretation and comparison with previous findings, the orientation distribution of boutons was converted into three equal categories. Accordingly, the proportion of boutons representing iso- (± [0–30] deg), oblique- (± [30–60] deg), and cross-orientation (± [60–90] deg) were calculated and displayed in Fig. 8c. Obviously, the majority of the connections (boutons) fell in the cross-orientation category (38%), while iso-orientation and cross-orientation connections represented an equal proportion (31%).

## Discussion

This study describes a new neuron type that shares morphological features of excitatory and inhibitory types in the 6th layer of the cat visual cortex. Neuron L6SC has a large cell body, and smooth spine-free arciform dendrites which are laden with input synapses. On the other hand, the axon displays features characteristic of excitatory neurons which establish type I synaptic contacts and project into the white matter. However, despite the very large lateral extent of the L6SC axon in the deep layers the type of clustering typical for long-range axons in granular and supra-granular layers was not present. Rather, the axon formed a linear distribution along major axon branches.

Pertinent to ask why only a single example of the new L6 neuron type is presented here. Among the many possible factors, we reckon, that due to the extremely wide horizontal extent of the axon which can rich a large population of deep-layer neurons requires only a low density of L6SC cell bodies at the population level. Subsequently, using the intracellular labelling approach the sampling rate of such a rare neuron type remains low.

### Previous studies

**Figure 9:**
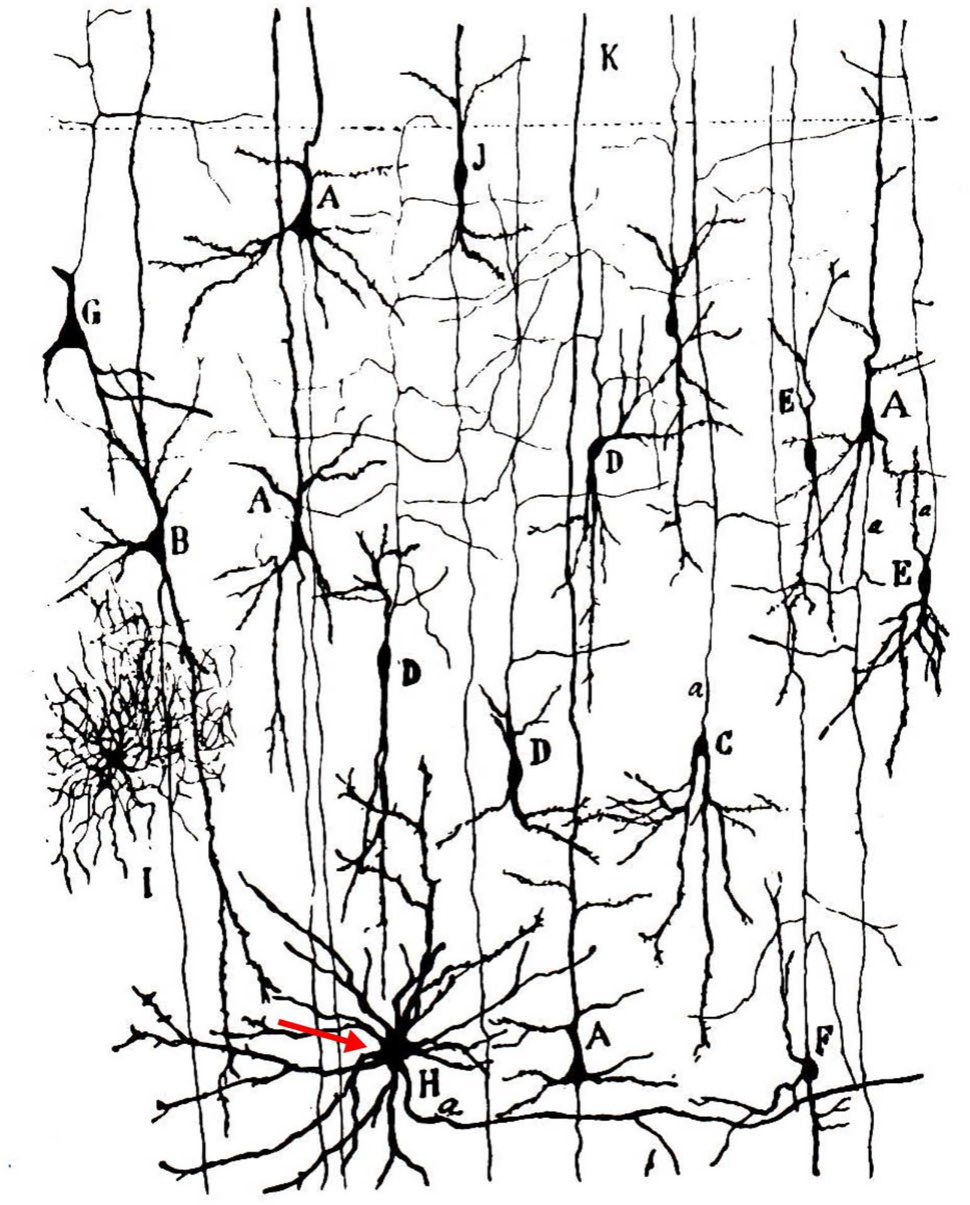
Modified drawing from the collection of Ramón’y Cajal showing Golgi-impregnated neurons in the motor cortex of the human infant. The neuron labelled with a red arrow in layer 7 is named “thick stellate cell with horizontal axon” (see page 230, Fig. 25 in^14^. Admittedly, by the above authors, “Although we have not seen the destination of the axon, we think it probable that these are cells with horizontal short axons, destined to establish associations at large distances.”

Layer 6 is named the multiform layer indicating the morphological opulence of neuron types such as pyramidal neurons and non-pyramidal neurons in terms of cell body shape, laminar location, orientation and connections with cortical and subcortical structures (for reviews^15,16^). The earliest morphological study of L6 neurons was made by Ramón y Cajal^14^ using the Golgi-impregnation technique. His seminal description of neuron types in different brain areas and species serves as a reference even today. Regarding the L6 stellate neuron type the closest description is probably the drawing of a cell body with non-spiny dendrites in the motor cortex of a human infant (Fig. 9). According to Cajal’s observation, the large stellate cell type has transverse or arciform recurrent axon collaterals in the deep layers and a descending main axon which is prolonged perhaps down to the white matter. The above description suits for the L6SC, however, without the structural subtleties which due to the inherent limitations of the Golgi method could not have been realized. As a matter of fact, Cajal did not report on the axons further than the major trunk and a few proximal shafts. He described a large cell body with spine-free dendrites. We reckon that the cell type described here might be the same but not beyond the shadow of a doubt since Cajal's giant neuron did not provide an 'arcade'-like dendritic field, a characteristic attribute of the L6 neuron described here. Based on recent studies carried out in mice^24^ and rats^10,25^ it should be noted that the closest equivalent of L6SC is a nonpyramidal neuron which is likely to target the basal dendrites of pyramidal neurons as well as the dendrites of non-pyramidal neurons of the same layer. A similar conclusion can be drawn from Golgi studies showing smooth dendritic interneurons in L6^26^ which were, nonetheless, different in a number of structural attributes from L6SC, such as synapse type and lacking white matter connection.

### Excitatory or inhibitory?

The present study aims to describe an L6 non-pyramidal neuron type which showed light- and electron microscopic features in an unusual constellation. While the smooth dendritic nature has been generally accepted for GABAergic neurons, asymmetric synapses are typically established by excitatory neurons. Since L6SC showed both of the above crucial features the question arises whether it is an inhibitory or excitatory neuron type. On the other hand, L6SC showed negative GABA-immunostaining (cell body, dendrites, boutons), a main axon entering the white matter and long-range horizontal axons which are rather characteristic for excitatory neurons than GABAergic. Taken together, it is just reasonable to assume that L6SC is an excitatory neuron type.

### Possible functional implications

Neurons of the deep layers have been considered to represent the major output to subcortical structures. In the visual cortex, L6 pyramidal cells are known to project in the visual thalamus^27^, superior colliculus^28^, and claustrum^29^. The L6SC main axon which entered the white matter may thus project towards the above-mentioned subcortical and/or cortical target areas. It should be also noted that, while L6SC targeted chiefly dendritic spines of presumed pyramidal neurons, 22% of the postsynaptic targets were dendritic shafts of which almost half contained GABA. These findings can be interpreted as L6SC is implicated in a circuitry to elicit both excitation as well as inhibition. Recent functional data showed that parvalbumin-containing neurons of L6 generate local as well as interlaminar inhibition^30^. It is tempting to ask whether the GABA-immunopositive dendrites postsynaptic to L6SC belong to which of the types, local or interlaminar. On the basis of axon morphology, a likely candidate could be the so-called L5 basket cell which was shown to possess long horizontal connections up to several millimeters in the deep layers. Indeed, there is a striking similarity between the distribution pattern of the axonal field of L6SC and L5 basket cells (see Fig. 1 in^31^). Long-range connections have been thought to generate long receptive fields and through this implicated in motion processing such as direction selectivity^32,33^. The intracortical connectivity features revealed here suit well for such a role of L6SC to be implicated in direction selectivity mechanisms^34,35^. On the other hand, L6SC receives a dense synaptic input on the dendrites. The integrative role that can be deemed from this feature is supported by the fact that L6SC has, indeed, three-times larger membrane surface area than any other neuron types examined so far (Table 2).

To take a more global view on the possible functional role of L6SC the available orientation map might be useful. Previous functional–anatomical studies demonstrated that long-range axons tend to terminate in regions of similar orientation preference to the parent soma^36–38^. It was also shown that this tendency is distance dependent, the further away from the soma the broader the orientation preference can be^19^ (for review, see^39^). In this regard, the L6SC soma was found near to an orientation center where many orientations converge. As expected the L6SC axon terminated in regions representing a broad range of orientations, mostly in regions representing non-iso-orientation. From such a functionally broad connectivity one can anticipate a role akin to normalization mechanisms (for rev., see^40^). Certainly, it remains to be seen whether L6SC is present in orientation domain zones with a similarly broad orientation preference of the axon.

## Conclusion and outlook

This study unravels the basic morpho-functional features of a novel neuron type, the L6 stellate cell (L6SC) in the primary visual cortex. While the cell body emits spine-free dendrites which is a characteristic feature of most GABAergic neurons L6SC was negative to GABA-immunostaining. Other features such as the main axon entering the white matter and the intracortical axon establishing asymmetric synapses chiefly with dendritic spines suggest that L6SC is an excitatory neuron type despite. The spatial extent of the L6SC axon and the rich innervation of the long and numerous dendrites indicate a signal integration role across remote cortical columns or at least to deal with properties which must be shared with neuron populations across large intracortical distances.

Finally, the classification of a neuron type is commonly based on several parameters including anatomical, physiological and molecular features^11^. The present work in identifying L6SC as a novel cell type was largely accounted for by morphological properties using light- and electron microscopy supplemented with GABA-immunohistochemistry. Obviously, numerous questions have remained unanswered, e.g. the frequency occurrence of L6SC in layer 6, topography and distribution of the axon in relation to other functional maps than orientation, firing properties and receptive field structure, neurotransmitter and neuromodulator content, just to mention a few. Thus, future work will be necessary for answering the above questions from which the functional significance of L6SC can be deciphered.

## Materials and methods

Animal experiments were performed according to the guidelines for the care and use of laboratory animals approved by the Animal Research Committee of the University of Debrecen (License Nr. 12/2016/DEMÁB) in accordance with the Hungarian Enactment for the Use of Laboratory Animals (40/2013.(II.14.) Gov.HUN) as well as complying with ARRIVE guidelines^41^ for the reporting of animal experiments.

### Surgery

For initial surgery, anesthesia was induced by intramuscular injection of a mixture of ketamine (7 mg/kg of CP-Ketamine 10%, Produlab Pharma B.V., SJ Raamsdonkseveer, The Netherlands) and xylazine (1 mg/kg of Primazin, Alfasan, JA Woerden, The Netherlands). Eye drops of 1.5% NaCl were used for protecting the corneal surface against drying out. Surgical wounds and pressure points were treated with Lidocaine (Xylocaine gel, EGIS Gyógyszergyár Zrt Budapest, Hungary). For arterial blood pressure measurement, a catheter (1.1 × 1.7 mm, B.Braun Melsungen AG, Germany) was inserted in the femoral artery while for artificial ventilation a Y-shaped cannula was implanted in the trachea. For muscle relaxation and nutrition, a so-called relax-mixture of Gallamine triethiodide (8 mg/kg/h Sigma-Aldrich Chemie GmbH, Steinheim, Germany) and glucose (24 mg/kg/h) diluted in Ringer solution was infused through the arterial catheter using a perfusor (Perfusor VI, B.Braun, Melsungen AG, Germany). Ten seconds after an initial dose of the relax-cocktail (4 ml/kg), artificial ventilation (Ugo-Basile cat/rabbit ventilator set for 10–13 strokes/min, 50–70 ml per stroke) commenced (N_2_O/O_2_ (70%/30%) supplemented with 0.3–0.6% isoflurane). Blood pressure (100–135 mmHg), end-tidal CO_2_ (3.8 ± 0.3%) and body temperature (38.5 °C) were continuously monitored throughout the experiment. Craniotomy was made in the posterior part of both hemispheres to expose the central visual representation of area 18 [Horsely–Clark coordinates, antero-posterior (AP): (− 5)–(+ 15), latero-medial (LM): (+ 0.5)–(+ 3.5)]^42^. A metal chamber (diameter, 30 mm) was fixed on the skull using dental cement (Paladur, Heraeus Kulzer, Wehrheim, Germany). Prior to optical imaging, the eyes were fitted with appropriate correction lenses to focus on the monitor screen (GDM-F520, Sony Electronics Inc., Park Ridge, NJ, USA) placed at 28.5 cm in front of the animal’s eye. For relaxing the nictitating membrane and for dilating the pupils, 5% phenylephrine-hydrochloride (Neosynephrin-POS 10%, Ursapharm, Saarbrücken, Germany) and 1% atropine sulfate (EGIS Gyógyszergyár Zrt Budapest, Hungary) were applied. During the experiments, eye positions were repeatedly checked on the basis of the tapetal back projection of retinal blood vessels.

### Optical imaging

Optical imaging was carried out to map orientation columns in the central visual representation of area 18 (Horsley–Clarke coordinates AP, 0–12 and LM, 0.5–3.5). Dura mater was removed and the chamber filled with Ringer solution that had been boiled in order to expel dissolved air. The vascular pattern of the cortical surface was imaged using green light (k = 545 ± 10 nm). During imaging of intrinsic signals, the cortex was illuminated with orange light (k = 609 ± 5 nm) using fibre-optic ring illumination fixed to the objective. Intrinsic signals were recorded through a CCD camera (BW model, PhotonFocus AG, Lachen SZ, Switzerland) attached to a ‘tandem-lens configuration’ (2 × SMC Pentax A 1:1.25, 50 mm). During data acquisition, the camera was focused 650 µm below the cortical surface. Images were acquired using the Imager 3001 and VDAQ software (Optical Imaging Inc., New York, NY, USA version 260). For generating visual stimuli the VSG Series Three (Cambridge Research System, Oxford, UK) system was used. Stimuli were presented on a CRT monitor (SONY, GDM-F520, 100 HZ, non-interlaced mode) placed at 57 cm in front of the cat’s eye. Visual stimuli consisted of high-contrast full-field square wave gratings at four equally spaced orientations that moved forth and back along the orthogonal axis of the orientation. The mean luminescence of the stimuli including the blank was 13 lux. Spatial and temporal frequency was optimized for area 18^28,43^ and each stimulus was presented 15 times in a pseudorandom manner. During the interstimulus interval lasting for 10 s, a stationary image of the next stimulus was presented. Data acquisition lasted for 4.5 s during which ten data frames were collected. The camera frames underwent spatial summation (two times binning) resulting in a spatial resolution of 16.129 µm per image pixel. Typically, gratings of 1 cycle per degree and 1 Hz temporal frequency yielded strong activity change and orientation domains characteristic for area 17^44–47^.

### Image analysis

Each block file consisted of a full set of activity images to all stimulus conditions. The organization of block files allowed the separation of the temporal sequences of image frames from which the development of the activity to each stimulus presentation could be monitored. Image analysis was done using the WinMix program (Optical Imaging Inc.) and custom-made programs written in IDL (Research Systems Inc., Colorado, CO, USA), and MATLAB (The MathWorks Inc., Natick, MA, USA). In the first step, image frames were extracted from the block files and single condition maps (SCM) generated. Then SCMs were normalized with the cocktail blank (the sum of all conditions including blank) followed by clipping of the images (this process is based on grey-scale statistics of image pixels whereby extreme values can be eliminated and the grey-scale range rescaled). For reducing low-frequency biological noise deriving from surface blood vessels and uneven illumination, box-car filters [high-pass (80 pixels) then low-pass (10 pixels)] were used for all images. The orientation angle map was calculated from the filtered SCMs using pixel-by-pixel vector summation and interpolation and displaying the results according to a chosen color scheme^48,49^.

### Intracellular injection

In order to stabilize recordings, artificial dura (AD) was prepared (silicone sheet, ShinEtsu Silicone Europe BV, Venlo, The Netherlands) which subtended the exposed cortical surface by 0.2 mm in each direction. At one location of the AD centered to the imaged cortex a 1 mm diameter aperture was made using a tissue sampling puncture (Harris Uni-Core, US Pat No 7093508). Then the AD was laid on the exposed cortex in a way that the subtending part was inserted between the dural edge of the craniotomy and the pia mater. Care was taken to avoid damaging blood vessels. Through the aperture an intracellular pipette filled with 1% KCl containing 2% biocytin was lowered on the cortex at an acute angle to the top of the lateral gyrus. The tip of the pipette, representing zero depth and the entry point was marked on the enlarged vascular image with 20 µm precision. Before driving the intracellular pipette (60–90 MΩ after beveling on a BV-10 M device, Sutter Instruments) in the cortex the chamber was filled with 3% agar and topped with low melting point paraffin (43 °C, Merck, Darmstadt, Germany). Signals were amplified using AxoClamp-2A (Axon Instruments, Foster City, Canada) in current-clamp mode while a change in the series resistance was monitored on the oscilloscope (0.1 nA step command and filtering at 10 kHz). After breaking in the neuron had − 56 mV resting membrane potential. Unfortunately, spontaneous activity was too high to determine the receptive field position. Nonetheless, intracellular labelling could be carried out using + 2 nA square wave current pulses (400 ms ON/200 ms OFF) for 11 min. Finally, for spatial alignment between the optical images and histological sections, 5 reference penetrations were placed along the rostro-caudal axis of the region of interest, i.e. in the vicinity of the intracellular recording site using empty glass micropipettes (~ 20 µm tip diameter whose entry points were marked on an enlarged printout of the vascular map) at stereotaxically determined locations^50^.

### Tissue fixation and histology

The animal received a lethal dose of anesthetics and was perfused transcardially with Tyrode’s solution (2 min) followed by 2 L of fixative containing 2% paraformaldehyde (VWR, Prolabo Chemicals, Leuven, Belgium) and 1% glutaraldehyde (Sigma-Aldrich Chemie GmbH, Steinheim, Germany) in 0.1 M phosphate buffer (PB, pH 7.4). From the tissue block containing the region of interest, 80 µm thick sections were cut using a vibratome (Leica S-1000, Wetzlar, Germany). Histology followed the protocol according to^21^. Briefly, sections were washed in PB (10 min) and 0.05 M Tris-buffered saline (TBS, 29 10 min) followed by avidin–biotin-complexed HRP (ABC-Elite, Vector, Burlingame, CA, USA) at 1:200 dilution in 0.05 M TBS containing 0.1% Triton X overnight at 4 °C. Next, the sections were washed in TBS (2 × 10 min), Tris-buffer (TB, pH 7.6, 10 min), and incubated in 0.05% of 3,3-diaminobenzidine (DAB, Sigma-Aldrich, St. Louis, MO, USA) in TB containing 0.0025–0.005% CoCl_2_. DAB was visualized by adding an equivalent amount of 1% H_2_O_2_ to the incubation medium to reach 0.01% final concentration. The reaction was terminated by an excess of TB in succession. All the sections were post-fixed in OsO_4_ (1% in 0.1 M PB, 45 min, Sigma-Aldrich), dehydrated in ascending series of ethanol followed by two times propylene oxide (Merck) and transferred in resin (Durcupan, Sigma-Aldrich) for 24 h. The next day the sections were mounted on slides, coverslipped and cured at 56 °C for 24 h^51^.

### Light microscopic reconstruction

The labelled cell was reconstructed using × 100 immersion oil objective and a Leica DMRB microscope attached to the Neurolucida (v.8.26) reconstruction system (MBF Bioscience Inc., Colchester, VT, USA). A total of 35 adjoining sections (60 µm) spanning the entire cortical depth and part of the white matter were aligned by using typically three pairs of cut ends of labelled neuronal processes, such as axons and dendrites, or fiducial landmarks such as small blood vessels. For this purpose, the align function of the Neurolucida program was used. In addition to this, section contours and reference penetrations, which resulted in a small tissue scar of about 20–80 µm in diameter, were also reconstructed. For matching the neuron reconstruction with optical images, the most superficial section containing the reference penetrations was used. In this way, a high-precision match could be achieved with a maximum alignment error of four image pixels or 64 µm^50^. Tissue shrinkage (× 0.8825) was determined as the ratio of reference penetration intervals measured in vivo and after histological processing. All morphological values were corrected for tissue shrinkage.

### Correlated light- and electron microscopy

Electron microscopic analysis was performed on representative samples of the labelled cell including cell body, dendrites and boutons. After thorough light microscopic documentation the selected structures were re-embedded in resin-filled beam-capsules using the protocol of^51^. Long series of 50 nm thick sections were cut and mounted on formwar-coated single-slot grids and contrasted with lead-citrate^52^. Synaptic specialization and postsynaptic structures to the labelled axon were identified in serial sections and identified as dendritic shaft or dendritic spine using ultrastructural criteria^8,53,54^.

### 3D-reconstruction of boutons

Serial EM sections of labelled boutons (n = 22) were photographed at × 40.000 magnification and reconstructed in 3D using the TrakEM2 plugin module of ImageJ software (https://imagej.nih.gov/ij/). Boutons of either *en passant* or with stalk (i.e. club-like) were reconstructed from 25 to 40 EM sections. Alignment of adjoining sections was made using red/green color view of the images under visual control until the best match was found (most yellow pixels). For volume and surface calculations, the reconstructed image stacks were exported in the Reconstruct program^55^. The Blender 2.92 software (GNU General Public License, https://www.blenderguru.com/) was used to visualize the reconstructed boutons.

### GABA-EM immunohistochemistry

Postsynaptic structures as well as the labelled cell were tested for GABA content. To this end EM sections containing the cell body, representative parts of dendrites, axons and boutons of the intracellularly labelled neuron were subjected using the colloidal-immunogold method^56^. Briefly, sections were mounted on formwar-coated single-slot gold grids. Each step was made on droplets (~ 50 µl each) of Millipore-filtered (0.22 µm) reagents placed on parafilm at room temperature. For avoiding contamination, the incubation was carried out in a Petri dish. First, the sections were treated with 1% periodic acid and 1% sodium periodate for 10 min (3 × 5 min DW wash in between) to etch the resin and remove osmium, respectively. Then the sections were washed 3 times in DW and once in 50 mM Tris-buffered saline (TBS, pH 7.4). For blocking the unspecific binding of the primary antiserum the sections were incubated in 1% aqueous solution of ovalbumin for 30 min, washed in TBS and put immediately on droplets of anti-GABA serum raised in rabbit (Sigma-Aldrich, A2052; diluted 1:1000 in TBS containing 1% normal goat serum) for 90 min. Next, the sections were washed in TBS for 2 × 10 min and incubated in 0.1% polyethylene glycol dissolved in 50 mM TBS for 10 min. The secondary antiserum, goat anti-rabbit immunoglobulin coupled to colloidal gold (15 nm, BBI Solutions, EM.GAR 15; diluted 1:40 in the latter buffer) was used for 90 min followed by rinses in distilled water. After immunostaining, the sections were treated with saturated uranyl-acetate for 20 min and contrasted with lead citrate for 10 min^52^. Quantitative analyses of the morphological parameters were extracted using NeuroExplorer (MBF Bioscience Inc., Colchester, VT, USA) routines.

### Bouton cluster analysis

The goal of the mean-shift clustering analysis was to unravel inhomogeneities in the spatial distribution of boutons. It is a centroid-based technique that updates centroid candidates to be the mean of points in a particular region. Part of the analysis is the post-processing stage whereby the candidate points are filtered to remove near-duplicates before determining the final centroids based on the bandwidth of the input. The bouton distribution of the L6SC was subjected to the mean-shift algorithm using the Python platform with a mean-shift library function from Scikit-learn^18^. Different bandwidths were used for exploring the clustering power of the algorithm and for comparison with distinct neuron types published earlier.

### Layering of the cortex

Cortical layers were determined from horizontal sections, i.e. cut parallel to the cortical surface. Therefore, for the laminar location of the cell body, dendrites and axon collaterals, only a limited set of layer-specific landmarks could be detected. Amongst them the presence of large layer 5 pyramidal cell bodies and the relatively sharp border between layer 6 and the white matter where the density of neurons drops considerably^57,58^. The contour of all sections spanning the entire cortical thickness was reconstructed. In this way, the laminar position of the reconstructed cell could be unequivocally determined.

## Supplementary Information

Supplementary Figure 1.

Supplementary Figure 2.

Supplementary Legends.

## Data Availability

The light- and electron microscopic data that support the findings of this study are available upon reasonable request from the corresponding author (ZK).
