# Supplementary figures and images for: Form, synapses and orientation topography of a new cell type in layer 6 of the cat’s primary visual cortex
Source: Sci Rep. 2022 Sep 14;12:15428. doi: 10.1038/s41598-022-19746-9 (PMC9474457; doi:10.1038/s41598-022-19746-9)

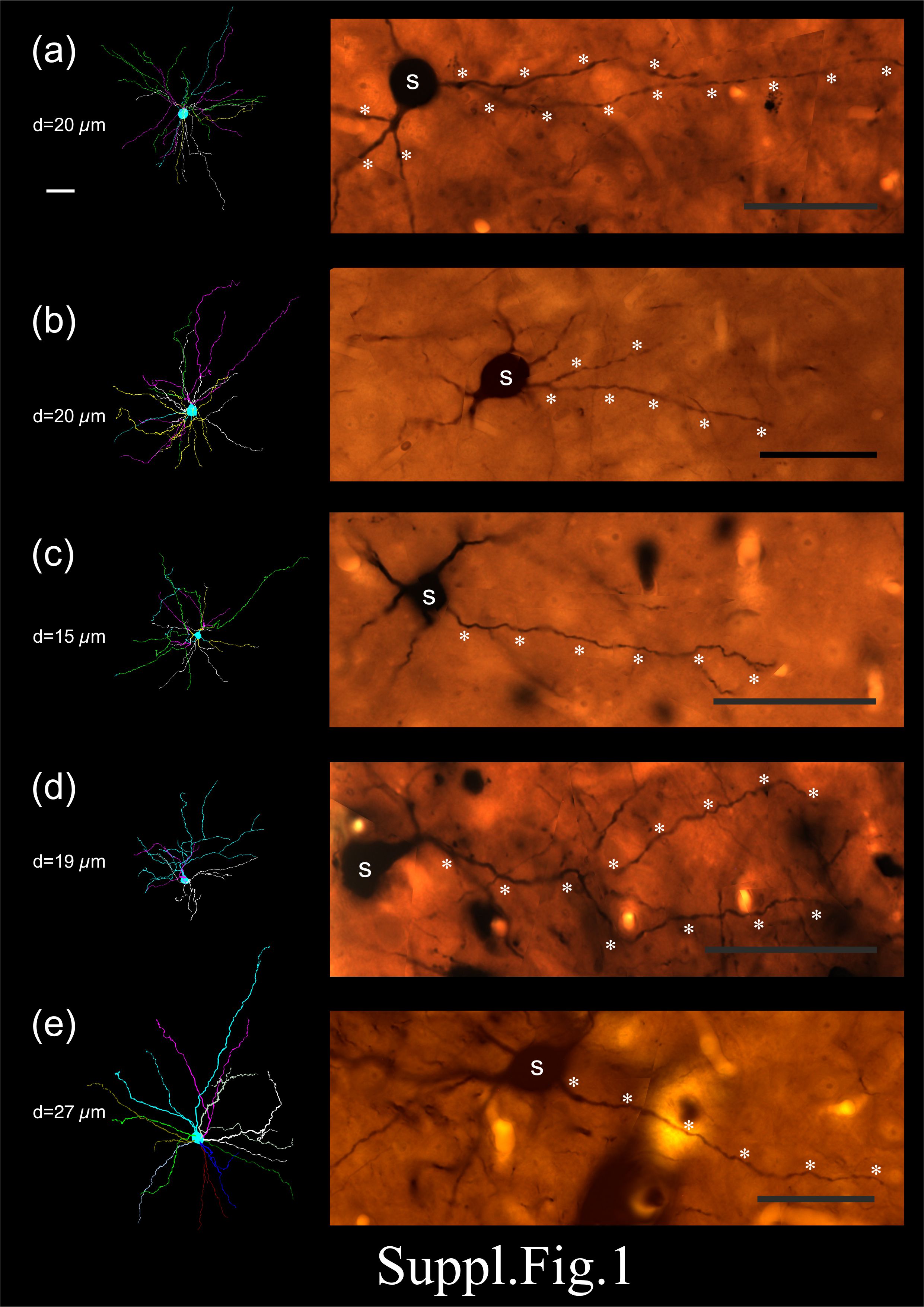

Supplement: Supplementary file 1 — Supplementary Figure 1. [file 41598_2022_19746_MOESM1_ESM.jpg]

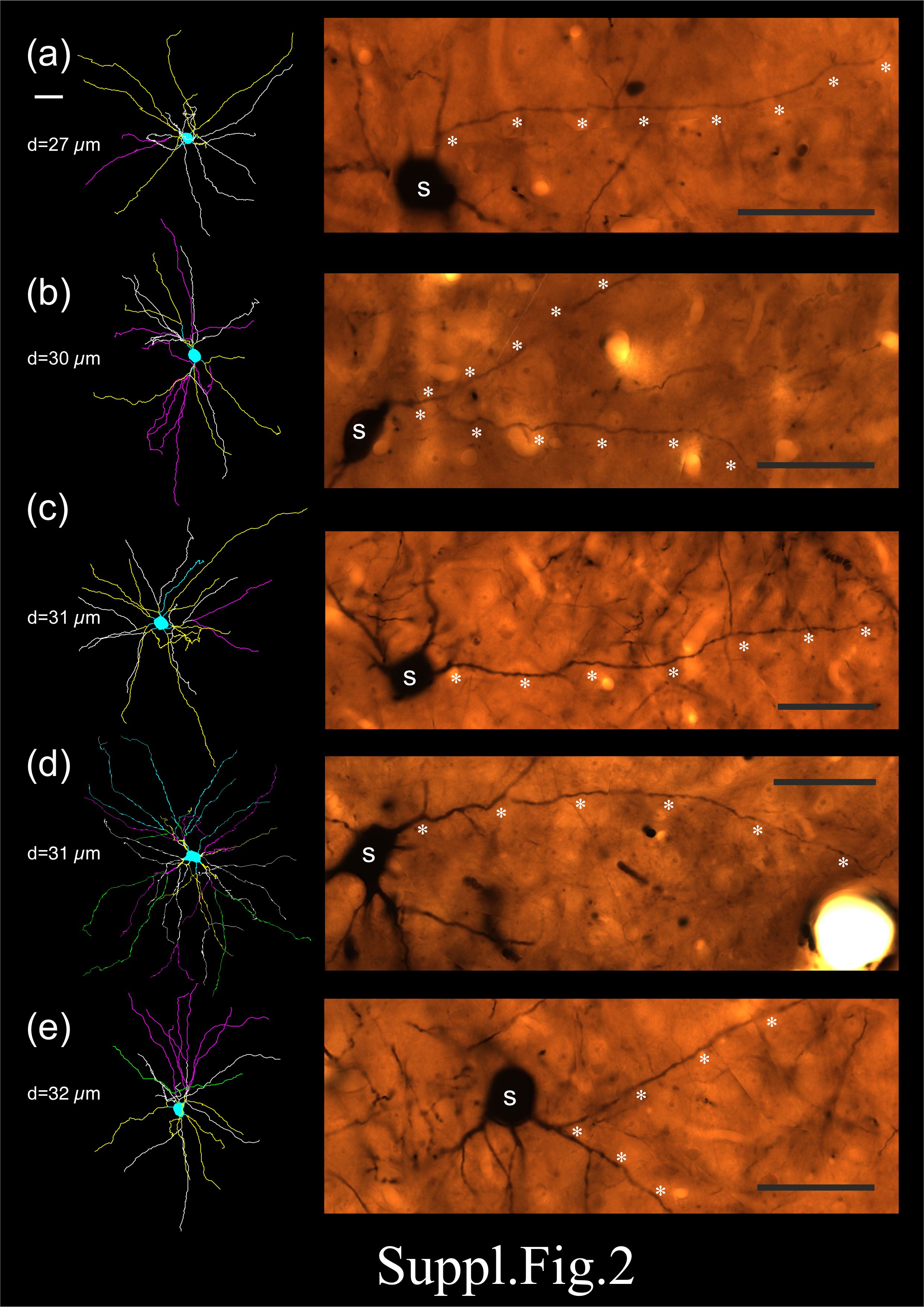

Supplement: Supplementary file 2 — Supplementary Figure 2. [file 41598_2022_19746_MOESM2_ESM.jpg]
