# Supplementary material for: Form, synapses and orientation topography of a new cell type in layer 6 of the cat’s primary visual cortex
Source: Sci Rep. 2022 Sep 14;12:15428. doi: 10.1038/s41598-022-19746-9 (PMC9474457; doi:10.1038/s41598-022-19746-9)
Supplement: Supplementary file 3 — Supplementary Legends. [file 41598_2022_19746_MOESM3_ESM.docx]

Legend to supplementary figures

Suppl.Fig.1

Light microscopic photo-montages of spine-free dendrites emerging from the soma (s) of 3D-reconstructed layer 3 medium size basket cells (a-c), layer 4 clutch cell (d), and layer 5 basket cell (e). Asterisks label the course of major dendrites often showing a beaded character. 3D-reconstructions of dendrites are shown on the left indicating soma diameter in micrometer. Drawings were created using the software package Neurolucida (v.8.26, MBF Bioscience, https://www.mbfbioscience.com). Scale bar (a-e): 50 µm.

Suppl.Fig.2

Light microscopic photo-montages of spine-free dendrites emerging from the soma (s) of 3D-reconstructed layer 3 large basket cells representing the largest smooth dendritic inhibitory neuron type (a-e). Asterisks label the course of major dendrites often showing a beaded character. 3D-reconstructions of dendrites are shown on the left indicating soma diameter in micrometers. Drawings were created using the software package Neurolucida (v.8.26, MBF Bioscience, https://www.mbfbioscience.com). Scale bar (a-e): 50 µm.
